# Applying cover crop residues as diverse mixtures increases initial microbial assimilation of crop residue‐derived carbon

**DOI:** 10.1111/ejss.13232

**Published:** 2022-03-25

**Authors:** Xin Shu, Yiran Zou, Liz J. Shaw, Lindsay Todman, Mark Tibbett, Tom Sizmur

**Affiliations:** ^1^ Soil Research Centre, Department of Geography and Environmental Science University of Reading Reading UK; ^2^ Soil Research Centre, Department of Sustainable Land Management, School of Agriculture, Policy and Development University of Reading Reading UK

**Keywords:** ^13^C‐PLFA, agroecology, arable soils, cover crops, decomposition, polyculture, priming, soil microbial biomass, soil organic matter, stable isotope probing

## Abstract

Increasing the diversity of crops grown in arable soils delivers multiple ecological functions. Whether mixtures of residues from different crops grown in polyculture contribute to microbial assimilation of carbon (C) to a greater extent than would be expected from applying individual residues is currently unknown. In this study, we used ^13^C isotope labelled cover crop residues (buckwheat, clover, radish, and sunflower) to track microbial assimilation of plant residue‐derived C using phospholipid fatty acid (PLFA) analysis. We also quantified microbial assimilation of C derived from the soil organic matter (SOM) because fresh residue inputs also prime the decomposition of SOM. To consider the initial stages of residue decomposition, and preclude microbial turnover, we compared a quaternary mixture of residues with the average effect of their four components 1 day after incorporation. Our results show that the microbial biomass carbon (MBC) in the treatment receiving the mixed residue was significantly greater, by 132% (3.61 μg C g^−1^), than the mean plant residue‐derived MBC in treatments receiving the four individual components of the mixture. However, there was no evidence that the mixture resulted in any additional assimilation of C derived from native SOM than the average observed in individual residue treatments. We surmise that, during the initial stages of crop residue decomposition, a greater biodiversity of residues increases microbial assimilation to a greater extent than would be expected from applying individual residues either due to faster decomposition or greater carbon use efficiency (CUE). This might be facilitated by functional complementarity in the soil microbiota, permitted by a greater diversity of substrates, reducing competition for any single substrate. Therefore, growing and incorporating crop polycultures (e.g., cover crop mixtures) could be an effective method to increase microbial C assimilation in the early stages of cover crop decomposition.

**Highlights:**

- The effect of mixing crop residues on assimilation of C by soil microbial biomass was investigated.
- The study is important due to recent interest in diverse cover crop mixtures for arable systems.
- Mixing crop residues enhanced the assimilation of plant residue‐derived C into microbial biomass.
- Growing and incorporating cover crop polycultures may enhance C storage in arable soils.

## 1 INTRODUCTION

Soil organic carbon (SOC) plays a critical role in global carbon (C) dynamics in the earth system and is a major property influencing soil functions and health. Applying crop residues to soils is a common strategy used in agroecosystems to enhance SOC stocks (Chapman & Newman, 2010; Chen et al., 2015). When microorganisms decompose plant residues and use the C for metabolism, they catabolise a portion of this C, which is usually released as carbon dioxide (CO_2_), and simultaneously assimilate and anabolise a portion of C into their biomass.

The fate of C after the application of plant residues of a single plant species is well understood (Rubino et al., 2010; Shahbaz et al., 2018). However, crop residues returned to soils under arable land management practices such as intercropping and cover crops include the residues of more than one plant species. Based on studies examining decomposition dynamics in soils receiving residue mixtures, it cannot necessarily be assumed that the application of a crop residue mixture will have the same impact on C dynamics as predicted from observations made on individual residues (Gartner & Cardon, 2004; Porre et al., 2020). If the behaviour or effect of a residue mixture can be predicted from the behaviour of the individual residues, this is classified as an additive effect. By contrast, a mixture could also deliver an antagonistic non‐additive effect (i.e., the mixture's effect is less than the average of individual species) or a synergistic non‐additive effect (i.e., the mixture's effect is greater than the average of individual species), which suggests there are interactions, via microbial decomposers, between the constituents of the mixture (Redin et al., 2014).

Previous studies exploring the effects of residue mixtures have focused on leaf litter decomposition and associated C cycling and nutrient release (e.g., nitrogen mineralization and immobilisation) in forest ecosystems (Castro‐Díez et al., 2019; Gartner & Cardon, 2004; Mao et al., 2017). However, the mechanisms responsible for non‐additive effects are not fully understood and might depend strongly on the context within which the study was conducted. To explain non‐additive effects, processes relating to nutrient transfer between nutrient‐rich (low carbon:nitrogen ratio, C:N) and nutrient poor (high C:N) litters, transfer of inhibitory compounds from one species' litter to another, or physical (water retention) effects have been frequently mentioned (Porre et al., 2020). In addition, mixing chemically contrasting residues may provide a greater number of niches for microorganisms to exploit, which allows functionally dissimilar microbial communities to coexist, and thus result in a greater microbial diversity and biomass than might be expected from the average of the individual communities that are supported by monocultures (Chapman & Newman, 2010). To better understand the mechanisms by which plant residue mixtures affect microbial assimilation of C, we need to track the fate of C supplied by individual components of the mixture and explore the potential for interactions between fresh plant residue inputs and older soil organic matter (SOM) via “priming effects”.

Priming effects are defined as short‐term changes in the turnover (i.e., microbial uptake and metabolism) of SOM caused by the input of easily degradable organic compounds (e.g., plant residues) to the soil by various mechanisms, including mining for nutrients (Kuzyakov, 2010; Kuzyakov et al., 2000; Wang et al., 2015). A priming effect is usually detected through measurement of respired CO_2_, as the end point of catabolism of SOM‐ and input‐C, and partitioning between sources using isotopic techniques. However, changes in SOM turnover linked to priming effects might also be manifest through the increased incorporation of native SOM‐C into microbial biomass on addition of fresh residue: increased availability, cellular uptake, and thus metabolic turnover of SOM constituents leads to increased SOM‐C incorporation to biomolecules through anabolic processes (Kuzyakov, 2010; Wang et al., 2015). Manipulating crop residue mixtures to maximise microbial C assimilation requires a consideration of the impact of amendments on native SOM turnover linked to microbial fate.

In this study, we investigated the residues of four functionally dissimilar crops from four different plant families (i.e., buckwheat, clover, radish, and sunflower) which are widely grown in mixtures as cover crops in agricultural systems. We established a microcosm experiment comprising treatments receiving either mixtures or individual (non‐mixture) ^13^C labelled cover crop residues which provided the same amount of residue‐derived C (1 mg C g^−1^ soil). Soil phospholipid fatty acid (PLFA) analysis was undertaken 1 day after incorporating crop residues to quantify the biomass of key soil microbial groups. We made our observations only 1 day after crop residues were applied to soils because we were interested in identifying the microbial groups which incorporate plant derived C directly into their biomass by anabolism, rather than the secondary turnover of C from microbial necromass, which could continue for months or years (Gunina & Kuzyakov, 2015). Gas chromatography‐combustion‐stable isotope mass spectrometry (GC‐C‐IRMS) was used to identify the microbial groups that had incorporated residue‐derived C and, by mass balance, quantify the amount of primed SOM‐derived C which was incorporated into the microbial biomass. We assumed that microbes have no preference for ^13^C over ^12^C. The difference between the mixture and the average of four non‐mixtures enabled us to determine whether the mixture delivered either a synergistic (mixture > average), an antagonistic (mixture < average), or an additive (mixture = average) effect.

We hypothesised that the mixture would result in a synergistic effect on the microbial assimilation of plant residue‐derived C because the mixture of crop residues increases the number of niches and provides a more diverse supply of nutrients, thereby creating conditions that facilitate the growth of a greater diversity microorganisms. Given that the cover crop species tested had divergent C:N ratios (ranging between 10 and 32 and spanning the threshold C:N [≈24]) for net N mineralization‐ immobilisation (Norton & Schimel, 2011), we hypothesised that adding residues in a mixture (average C:N = 17) would decrease the requirement for microorganisms to prime native SOM to scavenge for N and therefore induce an antagonistic effect on the microbial biomass C derived from primed SOM.

## 2 MATERIALS AND METHODS

### 2.1 Soil samples and crop residues

A silty loam Luvisol (World Reference Base classification); pH (H_2_O) 6.3, 22.32 g C kg^−1^, 2.24 g N kg^−1^, 0.90 mg NH_4_^+^ ‐N kg^−1^, 2.75 mg NO_3_^−^ ‐N kg^−1^ was collected from an arable field on the University of Reading's research farm at Sonning, Reading, UK (51.481152, −0.902188) in August 2019 after harvesting spring barley (*Hordeum vulgare*). Seven surface soil samples (0–20 cm depth) were randomly sampled and mixed thoroughly to create one homogenous sample, approximately 20 kg in weight.

Four cover crops, buckwheat (*Fagopyrum esculentum*), berseem clover (*Trifolium alexandrinum*), oil radish (*Raphanus raphanistrum*), and sunflower (*Helianthus annuus*), were continuously and uniformly labelled with ^13^CO_2_ in growth cambers by IsoLife (Wageningen, Netherlands). Buckwheat and clover were harvested 5 weeks after sowing, while radish and sunflower were harvested after 4 weeks. The ^13^C atom percent of the resulting aboveground biomass was 6.7%, 7.8%, 7.8%, and 8.0% for buckwheat, clover, radish, and sunflower, respectively. Corresponding unlabelled crops were grown under the same conditions in growth chambers by IsoLife (Wageningen, Netherlands), and harvested at the same time. After harvesting, the aboveground residues of both ^13^C labelled and unlabelled crops were dried at 70 °C and milled to pass through 0.05 mm mesh. The chemical composition of ^13^C labelled and unlabelled residues is provided in Table S1.

### 2.2 Experimental design

Field‐moist soil was sieved to pass a 4 mm mesh and then pre‐incubated for 7 days at 26 °C with soil water content at 60% of the water holding capacity (0.22 g g^−1^). As indicated in Table 1, the treatments consisted of pure unlabelled residues, labelled non‐mixture residues, quaternary mixtures of residues which contained one labelled species and three unlabelled species, and a control without any crop residue additions. For each treatment receiving residues, four replicate microcosms were established by mixing 150 g of fresh soil (equivalent to 122.95 g dry soil) thoroughly with a mass of dry residues to ensure C was added to each microcosm at a rate of 1 mg C g^−1^ soil in a plastic bag. In the pure treatments, all the added C was from the same unlabelled residue sample. In the non‐mixture treatments, 25% of added C was from the ^13^C labelled residue and 75% of added C was from the unlabelled residue of the same crop species. In the mixture treatments, 25% of added C was from a ^13^C labelled crop and 75% of added C comprised unlabelled residues from the other three crop species. A 100 g subsample of each fresh amended soil (equivalent to 78 g dry soil) was packed into bulk density rings (98 cm^3^) at a bulk density of 0.8 g cm^−3^ and incubated in darkness in a constant temperature room set to 26 °C in open‐top containers described by Adekanmbi et al. (2020).

**TABLE 1:** Experimental design and the C:N ratio of added crop residues in each treatment

Treatment	Plant	Abbreviation	Description	Added ^13^C amount (mg C g^−1^ soil)	C/N ratio
Non‐mix	Buckwheat	NB	25% labelled + 75% unlabelled buckwheat	0.0168	10
Non‐mix	Clover	NC	25% labelled + 75% unlabelled clover	0.0195	32
Non‐mix	Radish	NR	25% labelled + 75% unlabelled radish	0.0195	18
Non‐mix	Sunflower	NS	25% labelled + 75% unlabelled sunflower	0.0200	19
Mix	Buckwheat	MB	25% labelled buckwheat, 75% of unlabelled residues (clover, radish, sunflower)	0.0168	17
Mix	Clover	MC	25% labelled clover, 75% unlabelled residues (buckwheat, radish, sunflower)	0.0195	17
Mix	Radish	MR	25% labelled radish, 75% unlabelled residues (buckwheat, clover, sunflower)	0.0195	17
Mix	Sunflower	MS	25% labelled sunflower, 75% unlabelled residues (buckwheat, clover, radish)	0.0200	17
Pure	Buckwheat	PB	100% unlabelled buckwheat	\	9
Pure	Clover	PC	100% unlabelled clover		30
Pure	Radish	PR	100% unlabelled radish		21
Pure	Sunflower	PS	100% unlabelled sunflower	\	22
Soil			Soil only without any plant residue addition	\	\

*Note*: 25% and 75% refers to proportion of total added C. The quantity of C added was the same across all treatments (apart from the soil treatment), which was 1 mg C g^−1^ soil. The C:N (carbon:nitrogen) ratio in the mixture treatments were the average of four types of residues.

### 2.3 Phospholipid‐derived fatty acids (PLFA) extraction

One day after incubation with soil and crop residues, a 10 g aliquot of soil was sampled from each replicate and freeze‐dried for downstream analysis of PLFA. PLFA was extracted following the method described by Sizmur et al. (2011). Briefly, 4 g of freeze‐dried soil was extracted with 7.8 mL of Bligh and Dyer extractant containing chloroform: methanol: citrate buffer (1:2:0.8 v/v/v). The extracted phospholipids were methanolized as fatty‐acid methyl esters and dissolved in hexane for analysis by gas chromatography (GC).

### 2.4 Gas chromatography (GC)

PLFA methyl esters were analysed using an Agilent Technologies 6890 N gas chromatography equipped with a Supercowax 10 capillary GC column (60 m × 0.25 mm i.d. × 0.25 μm film thickness) and a Flame Ionisation Detector (FID). Helium was the carrier gas. The temperature programme was 1‐min isothermal at 60°C, followed by a ramp to 145°C at 25°C per minute, followed by an increase to 250°C at 2.5°C per minute and then held isothermally at 310°C for 10 min. Data were processed using GC ChemStation (Agilent Technologies). Peaks were identified using a bacterial fatty acid methyl esters (BAME) mix (Sigma Aldrich, UK) and quantified using a 37‐component fatty acid methyl esters (FAME) mix (Sigma Aldrich, UK). The biomass of each group of microorganisms was determined using the combined mass of fatty acids to which the group is attributed in Table S2.

### 2.5 Gas chromatography‐ combustion‐isotope ratio mass spectrometry (GC‐C‐IRMS)

GC‐C‐IRMS analysis was performed by injecting a 1 μL sample of fatty‐acid methyl esters into an Agilent 7890 N GC, upstream of a DELTA V™ Isotope Ratio Mass Spectrometer (electron ionisation, 100 eV, 1 mA electron energy, 3 F cup collectors m/z 44, 45 and 46, CuO/Pt Thermofisher GC IsoLink interface maintained at 1000°C). A Nafion membrane was used to prevent water from reaching the ion source. GC conditions were the same as that described above. Samples were calibrated against reference CO_2_ of known isotopic composition, which was introduced directly into the source five times at the beginning and end of every run. Data were processed in the Isodat Gas Isotope Ratio MS Software (ThermoFisher Scientific) to generate δ^13^C values representing the ratio of ^13^C/^12^C in fatty‐acid methyl esters, relative to the ^13^C/^12^C ratio of the international Pee Dee Belemnite (PDB) standard (0.01118).

δ^13^C values obtained for methylated compounds were corrected for the addition of derivative C following Zhang et al. (2019) and then the plant residue‐derived C, SOM‐derived C, and total C (including the C present in the PLFA biomass without residue addition; assessed by analysing the control treatment) were determined for the quaternary mixture and each non‐mixture treatment by following the equations outlined in the Data S1.

### 2.6 Data analysis

All the statistics were conducted in R (version 3.5.2) (R Core Team, 2018) except for the analysis of similarity (ANOSIM) which was conducted in Primer 7 (Primer‐e, Auckland, New Zealand). We used nested analysis of variance (ANOVA) to compare the effect from the quaternary mixtures with the effect from the average of the four non‐mixture treatments on total PLFA biomass C, the PLFA biomass C assimilated from crop residues, and the PLFA biomass C assimilated from SOM.

To compare the difference in microbial community structure between the mixture and the average of four individuals, PLFA data measured by GC‐FID was “Hellinger” transformed in all treatments. Non‐metric multidimensional scaling (NMDS) on Bray‐Curtis distance of the transformed data were performed using the “vegan” package in R (Oksanen et al., 2019). Bray‐Curtis distance similarity matrices were analysed by a one‐way analysis of similarity (ANOSIM) using Primer 7 to test if the differences between the mixture and the average of four non‐mixture treatments were significant.

## 3 RESULTS

### 3.1 Crop residues increased soil microbial biomass and altered community structure

The incorporation of cover crop residues significantly (*p* <0.001) shifted the microbial community structure away from the unamended control soil (Figure S1). The incorporation of residues from different crop species lead to significantly (*p* <0.05) different community structures (Figure S1). The biomass of general bacteria, Gram‐positive bacteria, and fungi in the unamended control soil was 1.80, 2.20, and 2.36 μg C g^−1^, respectively, which was greater than the biomass of Gram‐negative bacteria and protozoa (Table S3). Despite the same rate of C addition applied across all the treatments, total PLFA biomass differed between residue amendment treatments; ranging from 11.66 to 18.93 μg C g^−1^, which was significantly (*p* <0.05) greater than that in the control soil (Table S3).

### 3.2 Mixing crop residues increased total PLFA biomass and altered microbial community structure

**FIGURE 1:**
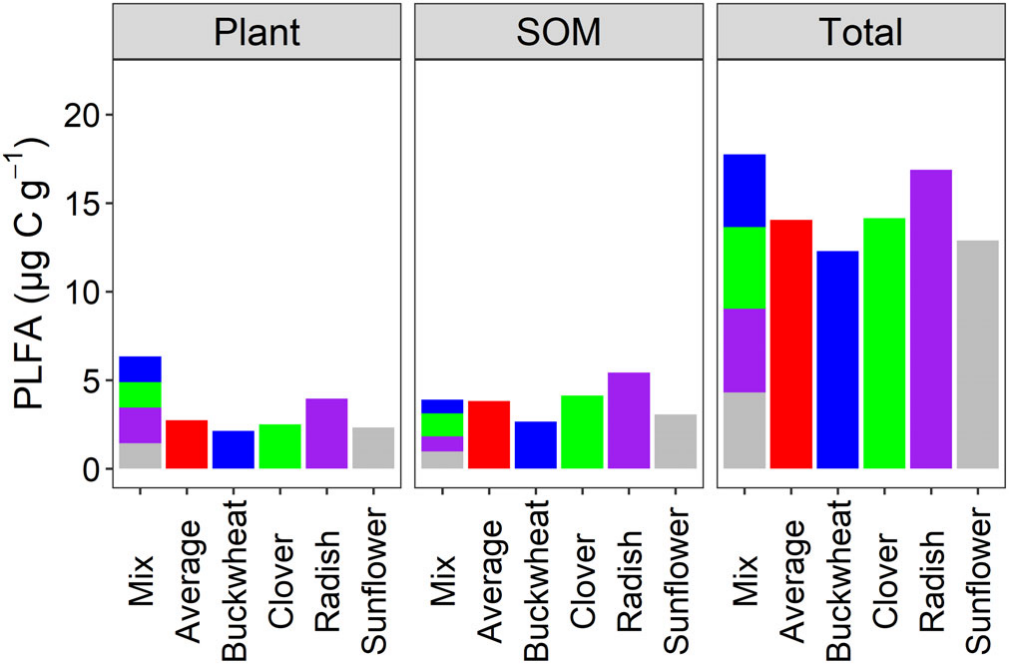
PLFA biomass carbon attributable to crop residues (plant), PLFA biomass carbon attributable to soil organic matter (SOM), and total PLFA biomass carbon (Total), in the mixture (mix), the average of four non‐mixture treatments (average), and the four individual non‐mixture treatments (buckwheat, clover, radish, and sunflower). The stacked bars in the mixture (mix) treatment shows the contribution of each cover crop (or SOM primed by the addition of each cover crop) to the PLFA biomass. The PLFA biomass in the (unamended) control soil was 7.50 μg C g^−1^ soil. Total PLFA was the sum of PLFA from plant, PLFA from SOM, and PLFA from control soil, where the PLFA from control soil was attributed equally to each cover crop species in the mixture (mix) treatment

Total PLFA biomass was 17.74 and 14.05 μg C g^−1^ for the mixture and the average of the four non‐mixture treatments, respectively (Figure 1). The soils in the mixture treatments had a significantly (*p* <0.05) greater total microbial biomass, by 26% (3.69 μg C g^−1^), compared to the soils in the non‐mixture treatments (Figure 1 and Table 2).

**TABLE 2:** Nested ANOVA (crop species nested within the non‐mixture treatment) to compare the effect of the mixture and the effect of the plant species on total PLFA‐C, crop residue‐derived PLFA‐C, and SOM‐ derived PLFA‐C of different microbial groups

Total PLFA‐C									
	DF	Sum of PLFA	General bacteria	G+ bacteria	G− bacteria	Fungi	Protozoa	G+/G−	Fungi/bacteria
Treatment	1	15.16**	9.16**	12.54**	0.07	9.66**	0.12	0.17	1.80
Treatment:Species	3	5.78**	2.13	9.55***	4.32*	2.69	0.95	3.46*	4.01*
Crop residue‐ derived PLFA‐C									
Treatment	1	20.62***	16.93***	4.64*	0.19	11.43**	0.22	0.40	0.02
Treatment:Species	3	1.36	0.12	1.34	2.66	1.41	0.85	0.71	0.4
SOM‐ derived PLFA‐C									
Treatment	1	0.01	0.07	1.33	0.00	0.03	0.01	0.44	0.53
Treatment:Species	3	2.72	2.14	3.97*	3.53*	1.48	0.81	1.08	2.63

*Note*: Treatment has two levels (mix and non‐mix). Crop species has five levels (radish, clover, buckwheat, sunflower, and mixture). DF is the degrees of freedom. Values are F values. *, **, and *** represent significance at *p* < 0.05, 0.01, and 0.001. G+ and G− bacteria are Gram‐positive bacteria and Gram‐negative bacteria, respectively. PLFA‐C, phospholipid fatty acid‐carbon; SOM, soil organic matter.

**FIGURE 2:**
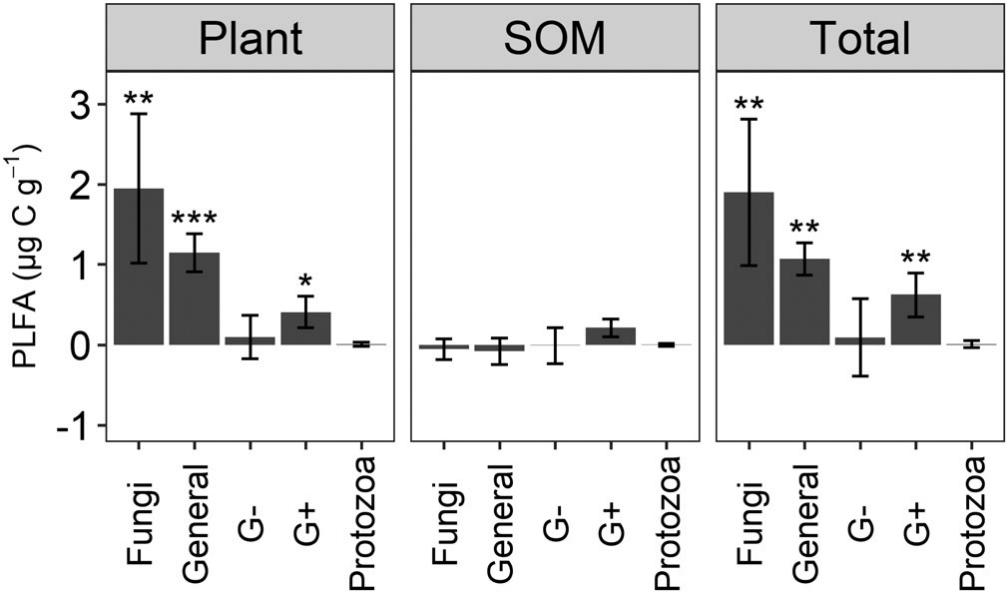
Differences in PLFA‐C (phospholipid fatty acid‐carbon) of key microbial groups between the mixture and the average of four non‐mixture treatments. Positive value means the mixture treatment had a greater biomass than the average of the non‐mixture treatments. The three panels represent PLFA biomass carbon attributable to crop residues (plant), PLFA biomass carbon attributable to soil organic matter (SOM), and total PLFA biomass carbon (Total). Error bars are standard deviations. General, G+, and G− represent general bacteria, Gram‐positive bacteria and Gram‐negative bacteria, respectively. *, **, or *** means significantly different from zero at the level *p* <0.05, 0.01, or 0.001

**FIGURE 3:**
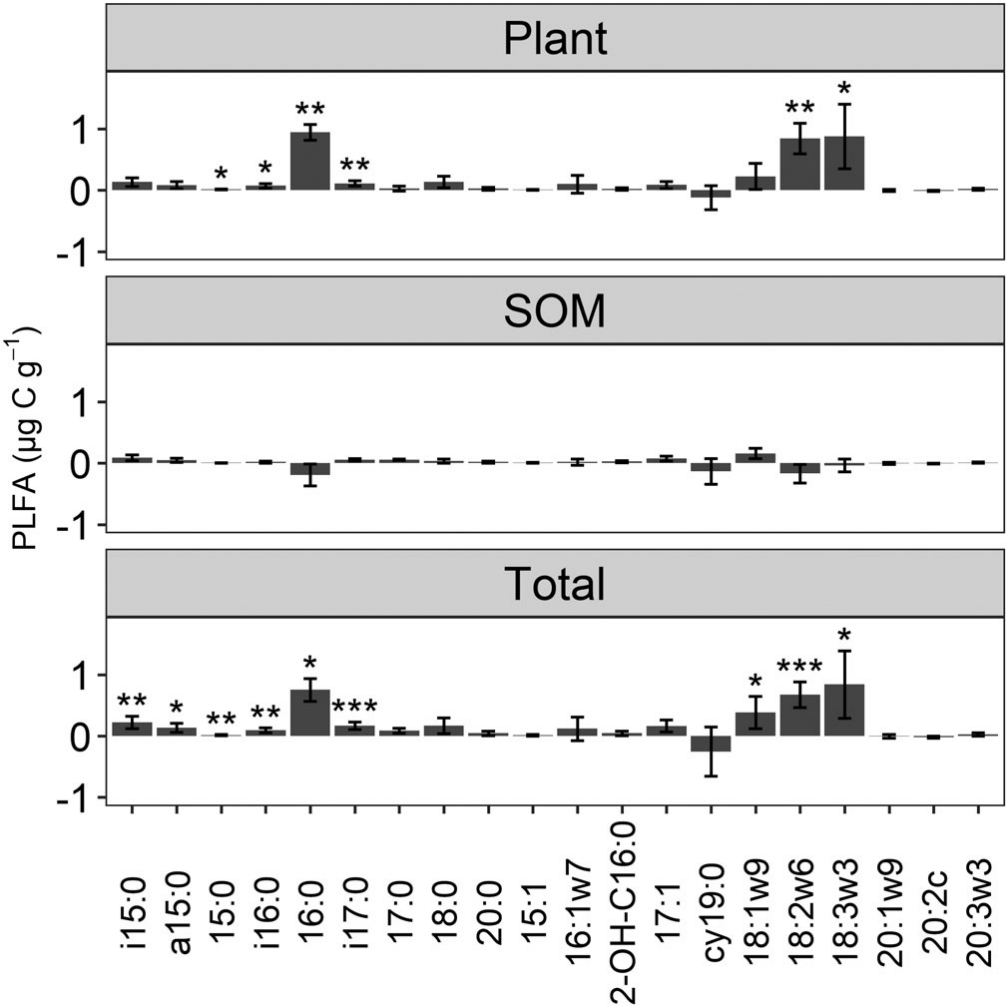
Differences in PLFA biomarker biomass between the mixture and the average of four non‐mixture treatments. Positive values indicate that the mixture has a greater biomass than the average of four non‐mixtures. The three panels represent PLFA biomass carbon attributable to crop residues (plant), PLFA biomass carbon attributable to soil organic matter (SOM), and total PLFA biomass carbon (Total). Error bars are standard deviations. *, **, and *** indicates significantly different from zero at the level *p* <0.05, 0.01, and 0.001, respectively

**FIGURE 4:**
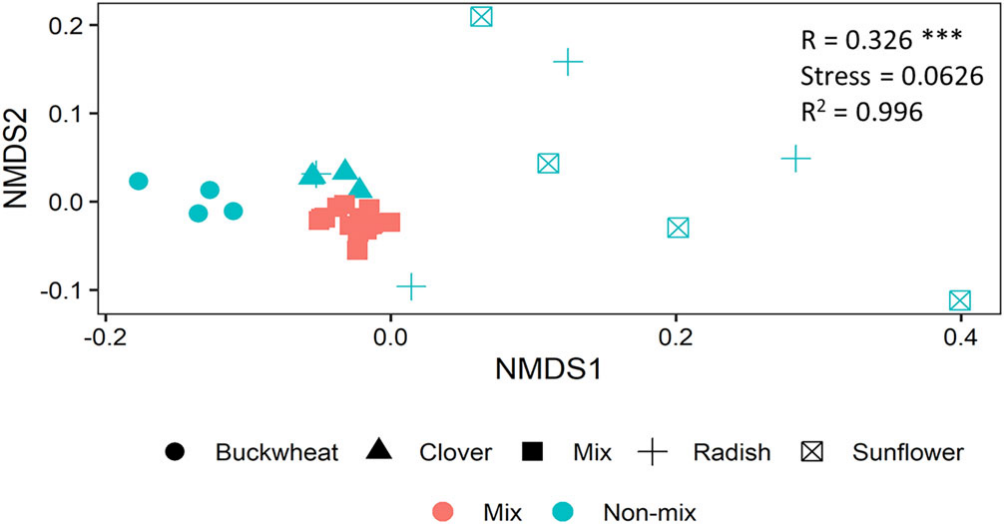
Microbial community structure in the mixture and non‐mixture treatments. Non‐metric multidimensional scaling (NMDS) on the bray‐Curtis distance on the Hellinger transformed PLFA data. Each symbol represents one sample. In the non‐mixture treatment, different shapes represent different crop species. R value followed by “***” indicates significant (*p* <0.001) difference between the mixture and the average of four non‐mixture treatments analysed by one‐way ANOSIM

The mixture treatment resulted in a significantly (*p* <0.001) greater general bacteria, Gram‐positive bacteria, and fungi biomass by 31% (1.07 μg C g^−1^), 18% (0.62 μg C g^−1^), and 38% (1.90 μg C g^−1^), respectively, than the average of the non‐mixture treatments (Figure 2 and Figure S4). In particular, biomarkers *i*15:0, 16:0, 18:1ω9, 18:2ω6, and 18:3ω3 were significantly (*p* < 0.05) more abundant in the mixture treatment than the average of four non‐mixture treatments (Figure 3). The ratios of fungi‐to‐bacteria in the mixture and the average of the four non‐mixture treatments were 0.64 and 0.57, respectively, which were not significantly different (Figure S2). Similarly, there was no significant difference in the ratio of Gram‐positive bacteria‐to‐Gram‐negative bacteria between the mixture and the average of four non‐mixture treatments (Figure S3). One‐way ANOSIM results demonstrated that microbial community structure in the mixture was significantly (*p* <0.001, R = 0.326) different from that in the average of the four non‐mixture treatments (Figure 4).

### 3.3 Mixing crop residues increased the microbial assimilation of residue‐derived carbon

Applying ^13^C labelled cover crop residues allowed us to distinguish the microbial biomass derived from plant residues from other resources. The results showed that the application of crop residues in the mixture and the average of four non‐mixture treatments resulted in 6.34 and 2.73 μg C g^−1^ PLFA‐C derived from crop residues, respectively (Figure 1). We observed significantly (*p* <0.05) greater microbial assimilation of crop residue‐derived C, by 132% (3.61 μg C g^−1^), in the mixture treatment, compared to the average of the four non‐mixture treatments (Figure 1).

The mixture exhibited a significantly (*p* <0.01) greater C derived from crop residues by general bacteria, Gram‐positive bacteria, and fungi by 193% (1.15 μg C g^−1^), 86% (0.41 μg C g^−1^), and 158% (1.95 μg C g^−1^), respectively, compared to the average of the four non‐mixture treatment (Figure 2, Figure S5, and Table 2). The biomass of 16:0, 18:2ω6, and 18:3ω3 in the mixture treatment were significantly (*p* <0.05) greater than those in the average of the four non‐mixture treatments (Figure 3). Based on the differences in PLFA biomass C derived from crop residues, there were no significant differences in either the fungi to bacteria ratio, or the Gram‐positive bacteria‐to‐Gram‐negative bacteria ratio, between mixture and the average of the four non‐mixture treatments (Figures S2 and S3).

### 3.4 Mixing crop residues did not increase microbial assimilation of SOM‐derived carbon

The PLFA‐C derived from primed SOM was the difference between total PLFA‐C, PLFA‐C in the control soil (7.50 μg C g^−1^ soil), and the PLFA‐C derived from crop residues. The results showed that the PLFA‐C derived from primed SOM was 3.90 and 3.82 μg C g^−1^ for the mixture and the average of four non‐mixture treatments, respectively (Figure 1). Therefore, we observed only 2% (0.08 μg C g^−1^) greater PLFA‐C derived from primed SOM between the mixture and the average of the four non‐mixture treatments, which was not statistically significant (Figure 1 and Table 2). None of the microbial groups, or the corresponding PLFA biomarkers, exhibited significant differences between the mixture and the average of the four non‐mixture treatments in terms of microbial assimilation of SOM‐derived C (Figure 2, Figure 3, and Figure S6).

## 4 DISCUSSION

Following the addition of ^13^C‐labelled residues, we compared the effect of mixing crop residues with the effect of applying the residues of a single crop species on the soil microbial community composition (Figure 4) and attributed the source of the C assimilated by the microbial biomass. Applying crop residues as a mixture resulted in significantly (*p* < 0.05) greater microbial biomass of crop residue‐derived C, compared to the average effect of applying each of the residues individually, indicating a synergistic effect of crop residue diversity on soil microbial assimilation (Figure 1). The mixture also exhibited greater total microbial biomass than any of the non‐mixture treatments (although not significantly greater in the case of radish; Figure S4), suggesting that a mixture of cover crop residues has the capability to increase soil microbial biomass more than the “best performing” monoculture.

The most common explanation for synergistic interactions during the decomposition of litter in a mixture is that N is transferred from low C:N residues to high C:N residues to satisfy microbial stoichiometric requirements (Gartner & Cardon, 2004; Mao et al., 2017). If the availability of N is the limiting factor in our experiment, we would expect to see the treatment receiving clover residues (which had the highest C:N ratio) exhibiting the smallest microbial biomass. On the contrary, the total microbial biomass in the treatment receiving clover residue (where C:N ratio was 32; Table 1) was significantly (*p* <0.05) greater than the treatment receiving buckwheat residue (where C:N ratio was 10; Table 1) (Figure S4), implying that N transfer between high C:N and low C:N residues to satisfy microbial stoichiometric requirement may not be the reason for the observed synergistic effects of the mixture. Since the soil contained 3.65 mg kg^−1^ mineral nitrogen prior to amendment, it is likely that there was sufficient available nitrogen to satisfy the microbial N requirement for the metabolism of the clover residues without the need to mineralise N from the low C:N ratio residues.

Since the four plant species used in this study come from four different plant families, their residues are likely to contain different plant secondary metabolites (e.g., tannins and terpenes) that suppress microbial resource assimilation (Gessner et al., 2010) or require induction of specialised enzymes for their degradation (Chomel et al., 2016). Thus, when mixing residues, each residue may provide a different ecological niche for decomposers. This increase in niches may have increased microbial biomass by allowing functionally dissimilar microbial populations to capitalise on residue C without competition, as evidenced by a microbial community composition in the treatment receiving a residue mixture that is different to the average of the treatments receiving single residues (Figure 4). A similar relationship has been observed between microbial biomass and root exudate diversity (Steinauer et al., 2016). Thus, we suggest that the non‐additive effect induced by the mixture was not predominantly controlled by residue bulk elemental composition (C:N ratio); instead, it could have been driven by the different chemical compositions of the different residues, including plant secondary metabolites, creating a greater number of ecological niches.

We found that the greater microbial biomass in soils receiving residue mixtures, compared to individual residues, can largely be attributed to C assimilated directly from the plant residues, rather than C obtained by enhanced priming of SOM (Figure 1). Microbial communities preferentially mineralise labile compounds after crop residues are applied to soils to build their biomass rather than decomposing pre‐existing SOM (Ball et al., 2014). Significantly greater crop residue‐derived C was observed in biomarkers 16:0, 18:2ω6, and 18:3ω3 in the soils amended with crop residue mixtures, compared to soils receiving individual residues (Figure 3). Although 16:0 is widely accepted as general bacterial biomarker and 18:2ω6 is widely used as a fungal biomarker, both are also found in plant tissues (Willers et al., 2015). Therefore, it is possible that these biomarkers represent plant biomolecules rather than microbial assimilation of plant‐derived C. However, if this was the case, we would expect to see the same abundance of these biomarkers in the mixture as we do in the average of the four individual residue treatments. We therefore assume that the greater abundance of these PLFAs in the mixture is due to greater microbial assimilation by bacteria and fungi. We found that fungi (biomarkers 18:2ω6 and 18:3ω3) were particularly efficient at assimilating C from a mixture of crop residues (Figure 3). This could be because fungal hyphae networks allow nutrients to be transported between microsites in the chemically and spatially heterogenous environment resulting from mixed residues (Ball et al., 2014). Nutrients other than N may be responsible for this synergy, especially since fungal communities have a lower N requirement than bacteria (Güsewell & Gessner, 2009). Furthermore, fungi can produce a wide range of extracellular enzymes that can degrade compounds which are recalcitrant for bacteria (Voriskova & Baldrian, 2013) and it has been previously observed that niche complementarity has a stronger influence on fungal communities than bacterial communities (Santonja et al., 2018).

Although the application of crop residues did induce microbial assimilation by priming the decomposition of native SOM, the magnitude of assimilation was not significantly changed by mixing crop residues (Figures 1 and 2), despite a different microbial composition (Figure 4). This observation was contrary to a previous assertion that mixtures of plant residues with a wide spectrum of labile compounds may support a higher microbial biomass and produce more extracellular enzymes, consequently enhancing the potential to prime the decomposition of recalcitrant compounds in SOM (Meier & Bowman, 2008). The lack of a marked mixture‐induced priming effect could, however, be because the amount of C added was the same in all treatments. A recent meta‐analysis, which analysed studies applying a range of organic C application rates, up to 3 mg C g^−1^, reported that the magnitude of the priming effect significantly increased with the increasing rate of additions, but was not affected by different residue types (Sun et al., 2019). If we had incubated our soils for longer, until available C was exhausted, then mixture treatments, which had a larger microbial biomass, may facilitate a stronger priming effect because of the increased need for nutrients to maintain microbial survival (Yu et al., 2020).

Our study revealed that the incorporation of crop residue mixtures increased the microbial C assimilation during the first day after residue application to a greater extent than would be expected by the addition of the same quantity of C from a single species residue. This additional microbial assimilation was mostly derived from the crop residues themselves, rather than primed SOM. It is not clear whether the reason for this greater microbial assimilation is due to faster metabolism (and thus anabolism) of the residues when in a mixture, or higher carbon use efficiency (CUE) by the microorganisms responsible.

There are limitations to the design of our study that constrain the interpretation of our data. The crop residues were dried and milled to 0.05 mm and mixed thoroughly with the soil. This preparation was necessary to ensure a homogenous distribution of the residues in the soil samples, but it results in physical shredding of the plant material that would not occur naturally and likely results in a greater proportion of the C becoming immediately soluble and available for microbial assimilation. Furthermore, the incubation period was very short (just 1 day). A longer incubation time may have provided greater insights regarding the ultimate fate of the C in the residues, but we would have been unable to distinguish primary decomposers from the secondary turnover of the C derived from the residues. Because the water‐soluble portions of plant materials are generally more rapidly decomposable than cellulose and lignin (Lee et al., 2011), the greater assimilation of these compounds in a mixture, as observed in this study, may not be representative of the assimilation of structural compounds that make up the majority of plant‐derived C.

## 5 CONCLUSION

Mixing of crop residues produced a synergistic effect on total soil microbial biomass because fungi, general bacteria, and Gram‐positive bacteria were able to assimilate crop residue‐derived C directly into their biomass to a greater extent than when applying individual crop residues. These findings may be due to a greater diversity of plant compounds providing more niches for microorganisms to exploit and subsequently a greater microbial diversity and biomass. Crop residue addition also stimulated the assimilation of native SOM into microbial biomass, but mixing residues resulted in an additive effect on microbial assimilation of SOM‐derived C. This might be facilitated by functional complementarity in the soil microbiota permitted by a greater diversity of substrates, reducing competition for any single substrate. Growing and incorporating a polyculture of crops (e.g., a cover crop mixture) may result in greater microbial C assimilation during the early stages of crop residue decomposition than observed when growing and incorporating monocultures.

## AUTHOR CONTRIBUTIONS

**Xin Shu:** Conceptualization (supporting); data curation (lead); formal analysis (lead); investigation (supporting); methodology (equal); project administration (supporting); visualization (lead); writing – original draft (lead); writing – review and editing (supporting). **Yiran Zou:** Data curation (supporting); formal analysis (supporting); investigation (supporting); methodology (equal); project administration (supporting); writing – review and editing (supporting). **Liz J. Shaw:** Conceptualization (supporting); investigation (supporting); writing – review and editing (supporting). **Lindsay Todman:** Conceptualization (supporting); formal analysis (supporting); investigation (supporting); writing – review and editing (supporting). **Mark Tibbett:** Conceptualization (supporting); investigation (supporting); writing – review and editing (supporting). **Tom Sizmur:** Conceptualization (lead); funding acquisition (lead); investigation (lead); methodology (equal); project administration (lead); resources (lead); supervision (lead); writing – original draft (supporting); writing – review and editing (lead).

## Supporting information


**Data S1** Supplementary information.

## Data Availability

Data sharing is not applicable to this article as no new data were created or analyzed in this study.
