# Supplementary material for: Applying cover crop residues as diverse mixtures increases initial microbial assimilation of crop residue‐derived carbon
Source: Eur J Soil Sci. 2022 Mar 25;73(2):e13232. doi: 10.1111/ejss.13232 (PMC9311145; doi:10.1111/ejss.13232)
Supplement: Supplementary file 1 — Data S1 Supplementary information. [file EJSS-73-0-s001.docx]

**Supporting Information for:**

**Applying crop residues as diverse mixtures increases microbial assimilation of plant residue-derived carbon**

**Xin Shu^1^, Yiran Zou^1^, Liz J. Shaw^1^, Lindsay Todman^2^, Mark Tibbett^2^, and Tom Sizmur^1^***

1. Soil Research Centre, Department of Geography and Environmental Science, University of Reading, UK.

2. Soil Research Centre, Department of Sustainable Land Management, School of Agriculture, Policy and Development, University of Reading, UK.

*Corresponding author – t.sizmur@reading.ac.uk +44(0)118 3788913

**This Supporting Information contains:**

**^13^C-PLFA calculations**

**3 Tables (Table S1, S2 and S3)**

**6 Figures (Figure S1, S2, S3, S4, S5, and S6)**

**13C-PLFA calculations**

GC-C-IRMS data was processed to generate δ^13^C values representing the ratio of ^13^C/^12^C in fatty-acid methyl esters, relative to the ^13^C/^12^C ratio of the international Pee Dee Belemnite (PDB) standard (0.01118) for all 13 treatments listed in Table S3.

δ^13^C values were obtained for methylated compounds where the methane group was added as part of the derivatisation. δ^13^C values were therefore corrected for the addition of derivative C using the mass balance approach described in equation 1 to 3, following Zhang *et al.*, (2019):

| $\delta^{13}C_{PLFA}(‰) = [(N_{PLFA} + 1) \times\delta^{13}C_{FAME}- \delta^{13}C_{MeOH}]/N_{PLFA}$ | (Eq 1) |
| --- | --- |
| $R_{sample} = (\delta^{13}C_{PLFA} / 1000 + 1) \times R_{PDB}$ | (Eq 2) |
| $F = R/(R+1)$ | (Eq 3) |

where δ^13^C_PLFA_, δ^13^C_FAME_, and δ^13^C_MeOH_, were the δ^13^C value of PLFA, FAME and methanol (δ^13^C_MeOH_ was assumed to equal zero), respectively. N_PLFA_ was the number of C atoms in this fatty acid to which the fatty acid methyl ester corresponds. R_sample_ and R_PDB_ were the ratios of ^13^C/^12^C in sample and in PDB. F is the fractional isotopic abundance representing the concentration of ^13^C as a proportion of the total concentration of C in the PLFA.

Equation 4 was used to calculate how much ^13^C (µg ^13^C g^-1^ soil) was incorporated into the PLFA biomass from the labelled residue in the non-mixture treatments:

| $13C incorporation (\mu g 13C / g soil) in non-mixture= F_{non-mixture} \times C_{non-mixture}$  $- F_{pure}\times C_{pure}$ | (Eq 4) |
| --- | --- |

F­_non-mixture_ and F_pure_ were the fractional abundances of ^13^C measured in the PLFA biomass of a non-mixture treatment and its corresponding pure unlabelled treatment, respectively. C_non-mixture_ and C_pure_ were the concentration of PLFAs (µg C g^-1^ soil) in the non-mixture and its corresponding pure unlabelled treatment, which were the results of GC-FID analysis. For example, ^13^C incorporation in the non-mixture of buckwheat (NB) equals to $F_{NB} \times C_{NB} - F_{PB}\times C_{PB}$. This step deducted the ^13^C incorporated into the PLFA biomass from the unlabelled residue and calculates only the ^13^C derived directly from the labelled residue.

For each mixture treatment, 75% of the added C was from three different unlabelled residues (25% from each residue). Similarly to equation 4, the ^13^C derived from these three unlabelled residues was deducted from the total ^13^C incorporated using equation 5 to obtain the ^13^C incorporated directly from the labelled residue (µg ^13^C g^-1^ soil).

| $13C incorporation (\mu g 13C / g soil) in mix= F_{mix}\times C_{mix}-0.25\times( F_{PB} \times C_{PB}+ F_{PC} \times C_{PC}+F_{PR}\times C_{PR}+F_{PS}\times C_{PS} )$ | (Eq 5) |
| --- | --- |

where F­_mix_ was the fractional abundances of ^13^C in the PLFA biomass of a mixture treatment. F_PB_, F_PC_, F_PR_, F_PS_, were the fractional abundances of ^13^C in the PLFA biomass of a pure unlabelled buckwheat, clover, radish, and sunflower treatment, respectively. C_mix_, C_PB_, C_PC_, C_PR_, C_PS_, were the concentration of PLFAs (µg C g^-1^ soil) in the treatments of mixture, pure buckwheat, pure clover, pure radish, and pure sunflower, which were analysed by GC-FID.

The proportion (unitless) of ^13^C incorporated from total amount of ^13^C added in the labelled residue was calculated using equations 6 and 7:

| $Total amount of added 13C (\mu g 13C / g soil)= Total added C \times Proportion of 13C labelled residue \times13C atom percent of labelled residue$ | *(Eq 6)* |
| --- | --- |
| \| $\mathrm{PLFA}proportion of added 13C= \frac{13C incorporation}{Total amount of added 13C}$ \| \| --- \| | *(Eq 7)* |

where total added C was 1000 µg C g^-1^ soil, the proportion of ^13^C labelled residue was 0.25. The ^13^C atom percent was 6.7%, 7.8%, 7.8% and 8.0% for buckwheat, clover, radish, and sunflower residues, respectively. This brings the total amount of ^13^C added in the labelled residue to 16.75, 19.50, 19.50, and 20.00 µg ^13^C g^-1^ soil, for treatments which received 25% of their added C from ^13^C labelled buckwheat, radish, clover, and sunflower.

^13^C is considered a tracer to track the amount of C derived from crop residues and incorporated into PLFA biomass. Assuming microorganisms have no preference for ^12^C over ^13^C, the proportion of PLFA incorporated ^13^C from added ^13^C should also be the same as the proportion of PLFA incorporated ^12^C from total added ^12^C. It is important to account for this since the labelled residues were not 100% ^13^C. Therefore, in all the non-mixture treatments, the amount of C incorporated into PLFA derived from the labelled crop residue C was calculated using equation 8:

| $\mathrm{PLFA}derived from\mathrm{labelled}residue in non-mixture (\mu g C/g soil)= PLFA proportion of added 13C * 1000 \mu g C/g soil$ | ((Eq 8) |
| --- | --- |

where 1000 µg C g^-1^ soil was the total added amount of C (equal to 1 mg C g^-1^ soil).

In the mixture treatment, the ^13^C labelled residue contributed 25% of the added C (i.e., 250 µg C g^-1^ soil). The proportion of ^13^C in each type of mixture (calculated by equation 7) was multiplied by 250 (µg C g^-1^ soil) to obtain the amount of PLFA biomass C derived from this ^13^C labelled crop. We then added up the amount of PLFA biomass C derived from each type of ^13^C labelled crop to determine the PLFA biomass C which was derived from the mixture of crop residues (equation 9).

| $\mathrm{PLFA}derived from residues in mixture (\mu g C/g soil)=({Proportion}_{MB}+{Proportion}_{MC}+{Proportion}_{MR}+{Proportion}_{MS}) * 250 \mu g C/g soil$ | (Eq 9) |
| --- | --- |

where proportion_MB_, proportion_MC_, proportion_MR_, and proportion_MS_ represent the proportion (unitless) of ^13^C incorporated from labelled ^13^C residues in each of the four types of mixture treatments where each type was amended with either ^13^C-labelled buckwheat, ^13^C-labelled clover, ^13^C-labelled radish, or ^13^C-labelled sunflower.

The PLFA biomass C derived from primed SOM was calculated as the difference between the total PLFA biomass C (determined by GC-FID), PLFA derived from residues (determined using equation 8 or 9), and PLFA in the control soil without any residue addition (determined using GC-FID) using equation 10.

| $\mathrm{PLFA}derived from SOM priming \left( \mu g C/g soil \right)$  $= total PLFA - PLFA derived from residues- PLFA in the soil only treatment$ | (Eq 10) |
| --- | --- |

where total PLFA and PLFA in the soil only treatment were determined by GC-FID and converted to µg C g^-1^ soil.

To determine the total PLFA biomass C in the mixture treatment, of which there were 16 experimental units across 4 different treatments, we averaged the PLFA biomass C (determined using GC-FID) from a single replicate of each of the 4 treatments and did this for each of the 4 replicates, resulting in four replicate measurements of total PLFA biomass C in the mixture, as calculated by equation 11.

| $\mathrm{Total}PLFA in mixture \left( \mu g C/g soil \right)$  $= 0.25*({total PLFA}_{MB} + {total PLFA}_{MC}+ {total PLFA}_{MR}+ {total PLFA}_{MS})$ | (Eq 11) |
| --- | --- |

Therefore, there were four replicate measurements of total PLFA biomass C, residue derived PLFA biomass C, and SOM derived PLFA biomass C that were used for statistical analysis with a balanced design.

1. The chemical composition of ^13^C labelled and unlabelled crop residues. The ^13^C atom percent in the unlabelled crops was not measured.

| Crop | Family | C (%) | | N (%) | C/N ratio | ^13^C atom percent (%) |
| --- | --- | --- | --- | --- | --- | --- |
| **^13^C labelled plants** | | | | | | |
| Buckwheat | Polygonaceae | 38.77 | | 2.73 | 14 | 6.7 |
| Clover | Fabaceae | 40.70 | | 1.00 | 41 | 7.8 |
| Radish | Brassicaceae | 35.96 | | 2.73 | 13 | 7.8 |
| Sunflower | Asteraceae | 37.46 | | 2.65 | 14 | 8.0 |
| **Unlabelled plants** | | |  |  |  |  |
| Buckwheat | Polygonaceae | 37.12 | | 4.28 | 9 | \ |
| Clover | Fabaceae | 39.30 | | 1.28 | 31 | \ |
| Radish | Brassicaceae | 37.19 | | 1.76 | 21 | \ |
| Sunflower | Asteraceae | 38.48 | | 1.66 | 23 | \ |

1. The attribution of PLFA biomarkers to microbial groups

| Biomarkers | Microbes | Reference |
| --- | --- | --- |
| i15:0 | Gram-positive bacteria | (Zelles, 1999)  (Zelles, 1999)  (Zelles, 1999)  (Zelles, 1999)  (Zelles, 1999) |
| a15:0 | Gram-positive bacteria |  |
| i16:0 | Gram-positive bacteria |  |
| i17:0 | Gram-positive bacteria |  |
| 15:1 | Gram-negative bacteria |  |
| 16:1w7 | Gram-negative bacteria | (Zheng *et al.*, 2018) |
| 17:1 | Gram-negative bacteria | (Zelles, 1999) |
| cy19:0 | Gram-negative bacteria | (Zelles, 1999) |
| 2-OH-C16:0 | Gram-negative bacteria | (Zelles, 1999) |
| 18:2w6 | Fungi | (Zheng *et al.*, 2018) |
| 18:1w9 | Fungi | (Zheng *et al.*, 2018) |
| 18:3w3 | Fungi | (Mbuthia *et al.*, 2015) |
| 20:1w9c | Fungi | (Zelles, 1999) |
| 20:2c | Protozoa | (Buyer & Sasser, 2012) |
| 20:3w3 | Protozoa | (Jin & Evans, 2010) |
| 15:0 | General bacteria | (Peacock *et al.*, 2001) |
| 16:0 | General bacteria | (Peacock *et al.*, 2001) |
| 17:0 | General bacteria | (Peacock *et al.*, 2001) |
| 18:0 | General bacteria | (Peacock *et al.*, 2001) |
| 20:0 | General bacteria | (Peacock *et al.*, 2001) |

1. Total microbial biomass (µg C g^-1^) in all treatments measured by GC-FID. Total is the sum of all the four microbial groups. G+ and G- are Gram-positive and Gram-negative bacteria. F/B ratio and G+/G- ratio represent the fungi to bacteria ratio, and the Gram-positive to Gram-negative bacteria ratio. Mean ± standard deviation (n = 4). *, **, and *** mean significant difference from the control soil at the level *P* < 0.05, 0.01 and 0.001, respectively. Refer to Table 1 for treatment codes.

| Treatment | General bacteria | G+ bacteria | G- bacteria | Fungi | Protozoa | Total | F/B ratio | G+/G- ratio |
| --- | --- | --- | --- | --- | --- | --- | --- | --- |
| MB | 4.13±0.59*** | 3.73±0.51*** | 2.20±0.36 | 6.26±0.86*** | 0.08±0.02 | 16.40±2.32*** | 0.62±0.02 | 1.70±0.06 |
| MC | 4.69±0.55*** | 4.09±0.41*** | 2.25±0.29* | 7.30±0.82*** | 0.09±0.01 | 18.42±1.77*** | 0.66±0.03* | 1.84±0.32 |
| MR | 4.76±0.48*** | 4.26±0.52*** | 2.43±0.31** | 7.38±0.73*** | 0.09±0.01 | 18.93±1.99*** | 0.65±0.03* | 1.75±0.03 |
| MS | 4.33±0.32*** | 3.90±0.30*** | 2.23±0.16* | 6.67±0.41*** | 0.09±0.01 | 17.22±1.14*** | 0.64±0.02* | 1.75±0.01 |
| NB | 3.06±0.19 | 2.79±0.16 | 1.41±0.10 | 4.99±0.59** | 0.03±0.04 | 12.29±1.04* | 0.69±0.05** | 1.99±0.05 |
| NC | 3.51±0.32** | 3.53±0.31*** | 1.89±0.17 | 5.12±0.30** | 0.08±0.01 | 14.14±1.09*** | 0.57±0.02 | 1.86±0.01 |
| NR | 4.02±0.61*** | 3.94±0.53*** | 2.77±0.76*** | 6.04±2.18*** | 0.10±0.12 | 16.88±3.35*** | 0.57±0.18 | 1.48±0.31 |
| NS | 3.05±1.20 | 3.22±0.24* | 2.68±1.16** | 3.86±0.82 | 0.08±0.04 | 12.89±0.57** | 0.44±0.12 | 1.40±0.60 |
| PB | 3.11±0.48 | 2.64±0.41 | 1.42±0.21 | 4.57±0.50* | 0.03±0.04 | 11.77±1.48* | 0.64±0.10* | 1.86±0.02 |
| PC | 3.02±0.27 | 2.99±0.13 | 1.54±0.06 | 4.51±0.27* | 0.02±0.03 | 12.08±0.71* | 0.60±0.01 | 1.94±0.03 |
| PR | 3.69±0.41*** | 3.69±0.33*** | 2.06±0.21 | 5.36±0.40** | 0.05±0.03 | 14.84±1.39*** | 0.57±0.02 | 1.79±0.02 |
| PS | 2.96±0.36 | 2.74±0.35 | 1.46±0.17 | 4.48±0.60* | 0.03±0.04 | 11.66±1.49* | 0.62±0.02 | 1.88±0.05 |
| Soil | 1.80±0.27 | 2.20±0.27 | 1.15±0.14 | 2.36±0.32 | 0.00±0.00 | 7.50±0.99 | 0.46±0.01 | 1.92±0.03 |


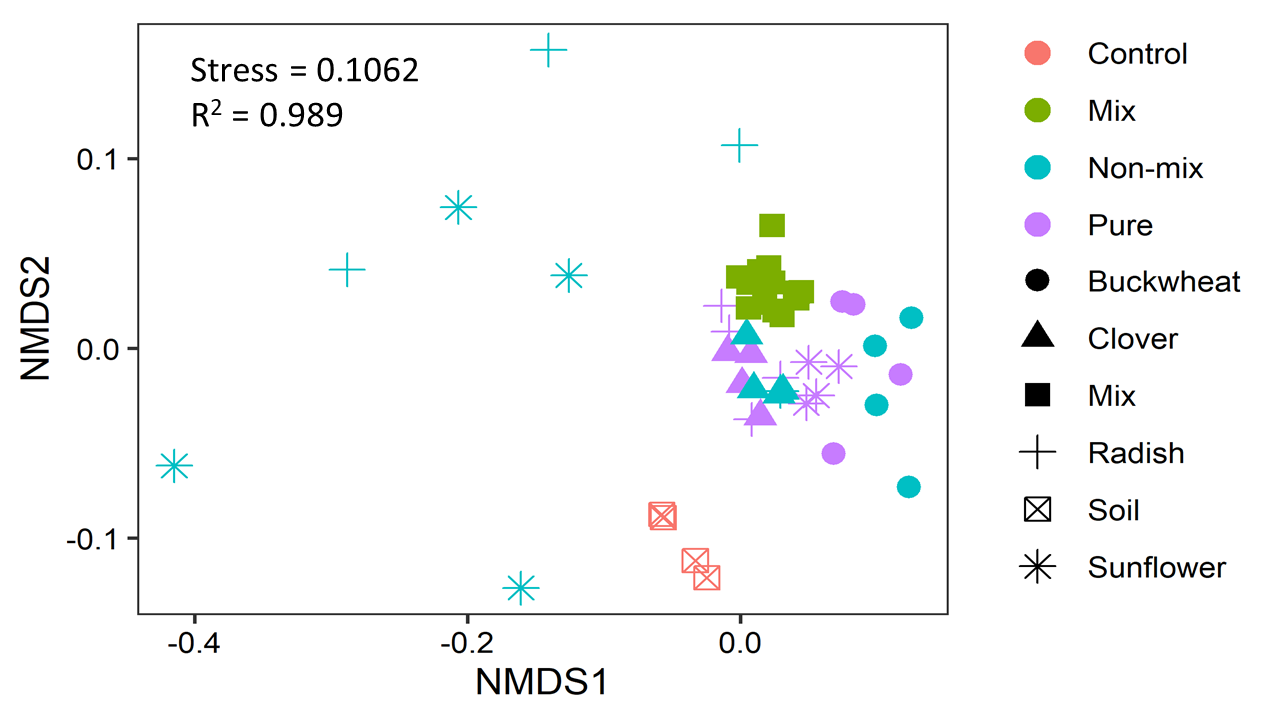


1. Bacterial community structure. Non-metric multidimensional scaling (NMDS) on the Bray-Curtis distance on the Hellinger transformed PLFA data. Dots the NMDS scores of samples. For the pure and non-mixture treatment, different shape represents the plant species in that treatment. One-way ANOSIM indicates a significant (R = 0.899, *P* < 0.001) difference between all the pure and control treatments. In the pure treatments, different plant species induced significant (*P* < 0.05) different microbial community composition.


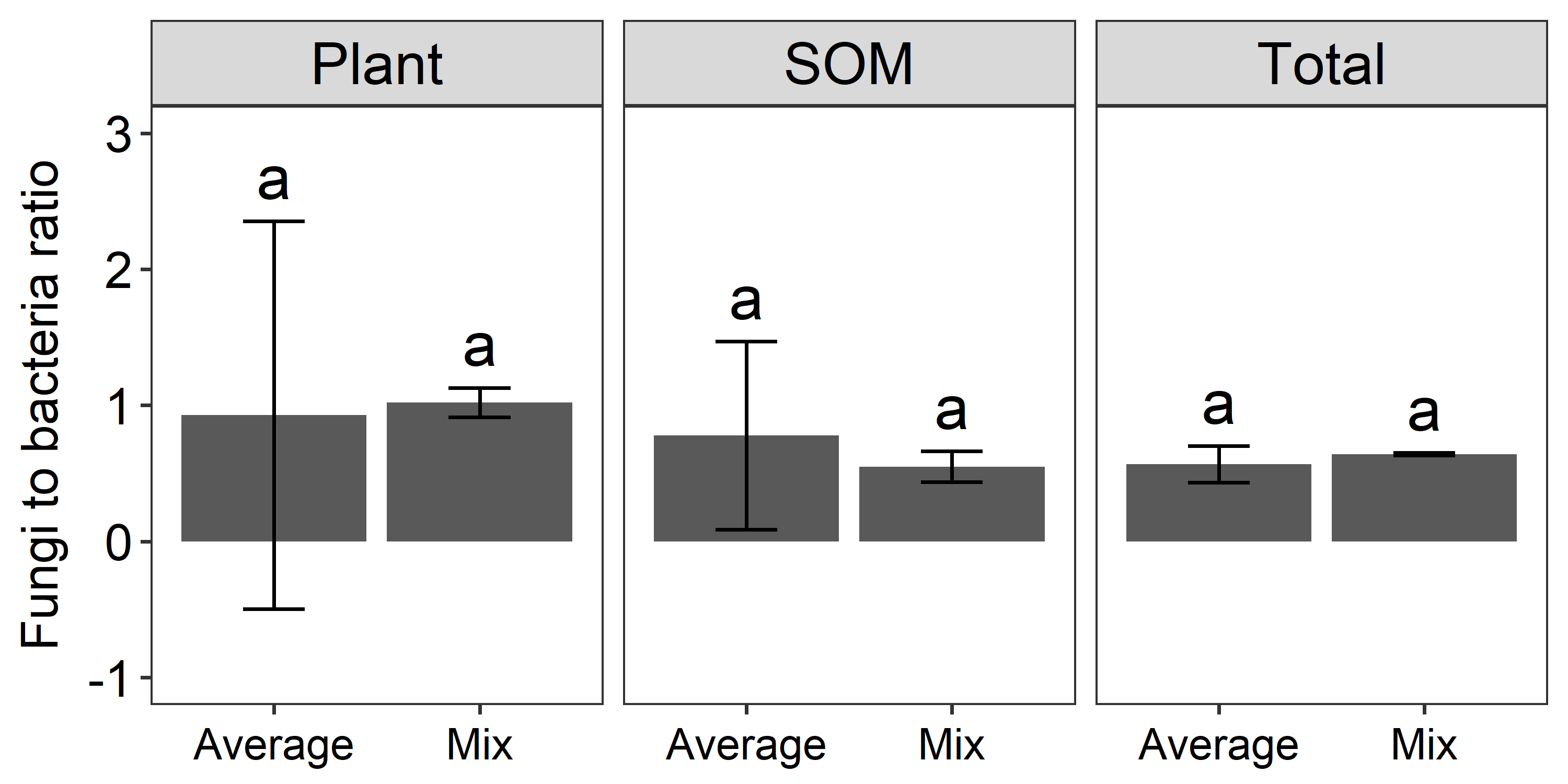


1. The ratio of fungi to bacteria biomass based on the PLFA derived from crop residues (Plant), primed soil organic matter (SOM), and total PLFA (Total). Average is the average of four non-mixture treatments. Different letters within the same panel mean significant difference between mixture and the average of four non-mixture treatments at *P* < 0.05. The error bars are standard deviations (n = 4).


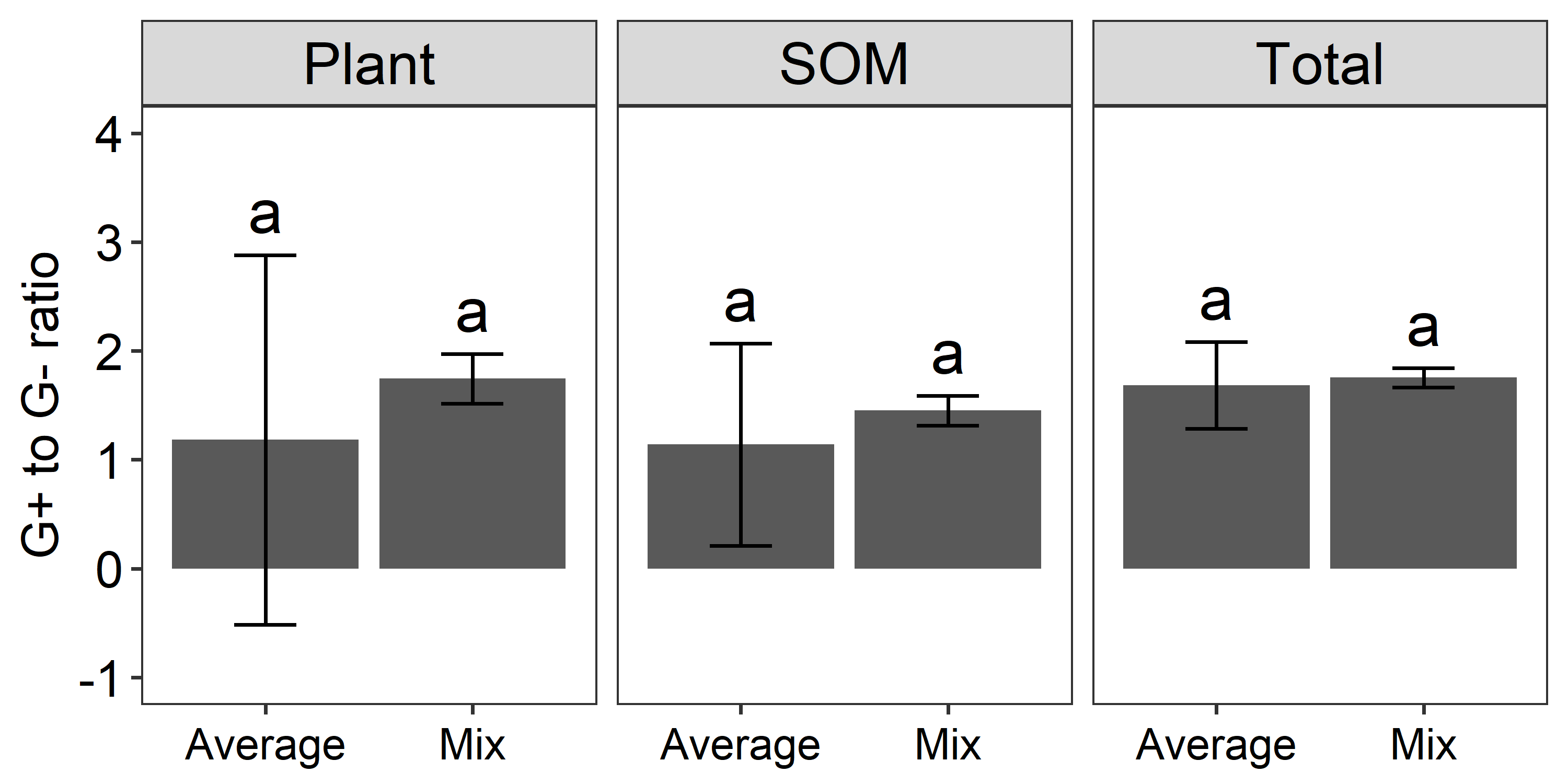


1. The ratio of Gram-positive bacteria (G+) to Gram-negative bacteria (G-) biomass based on the PLFA derived from crop residues (Plant), primed soil organic matter (SOM), and total PLFA (Total). Average is the average of four non-mixture treatments. Different letters within the same panel mean significant difference between mixture and the average of four non-mixture treatments at *P* < 0.05. The error bars are standard deviations (n = 4).


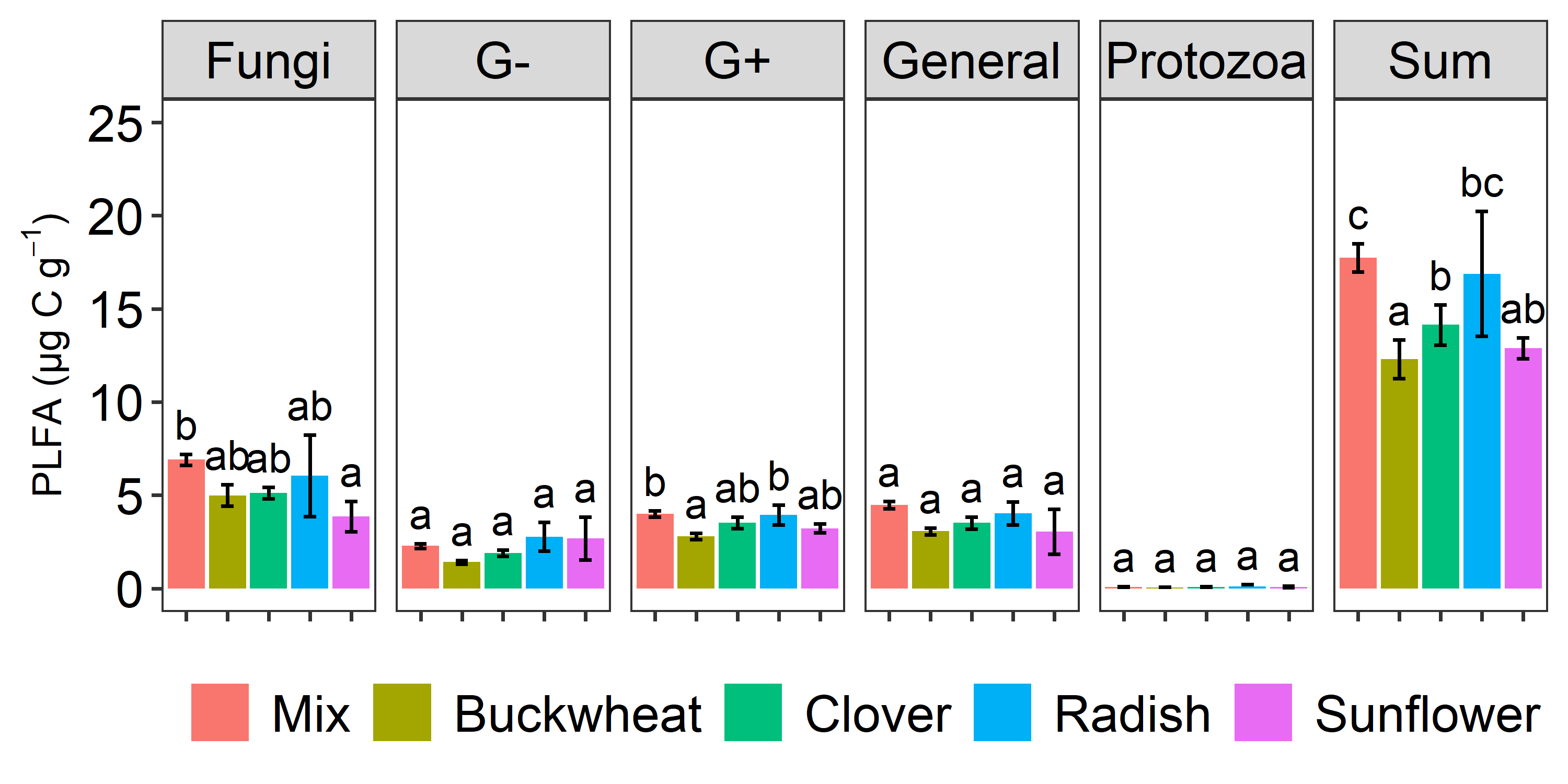


1. Total PLFA biomass in the mixture, and non-mixture (buckwheat, clover, radish, and sunflower) treatments. Sum is the sum of microbial biomass including all the microbial groups. G+, G-, and General represent Gram-positive, Gram-negative, and general bacteria. Error bars are standard deviations (n = 4). Different letters above bars indicate significant differences in PLFA biomass between treatments for that microbial group at *P* < 0.05.


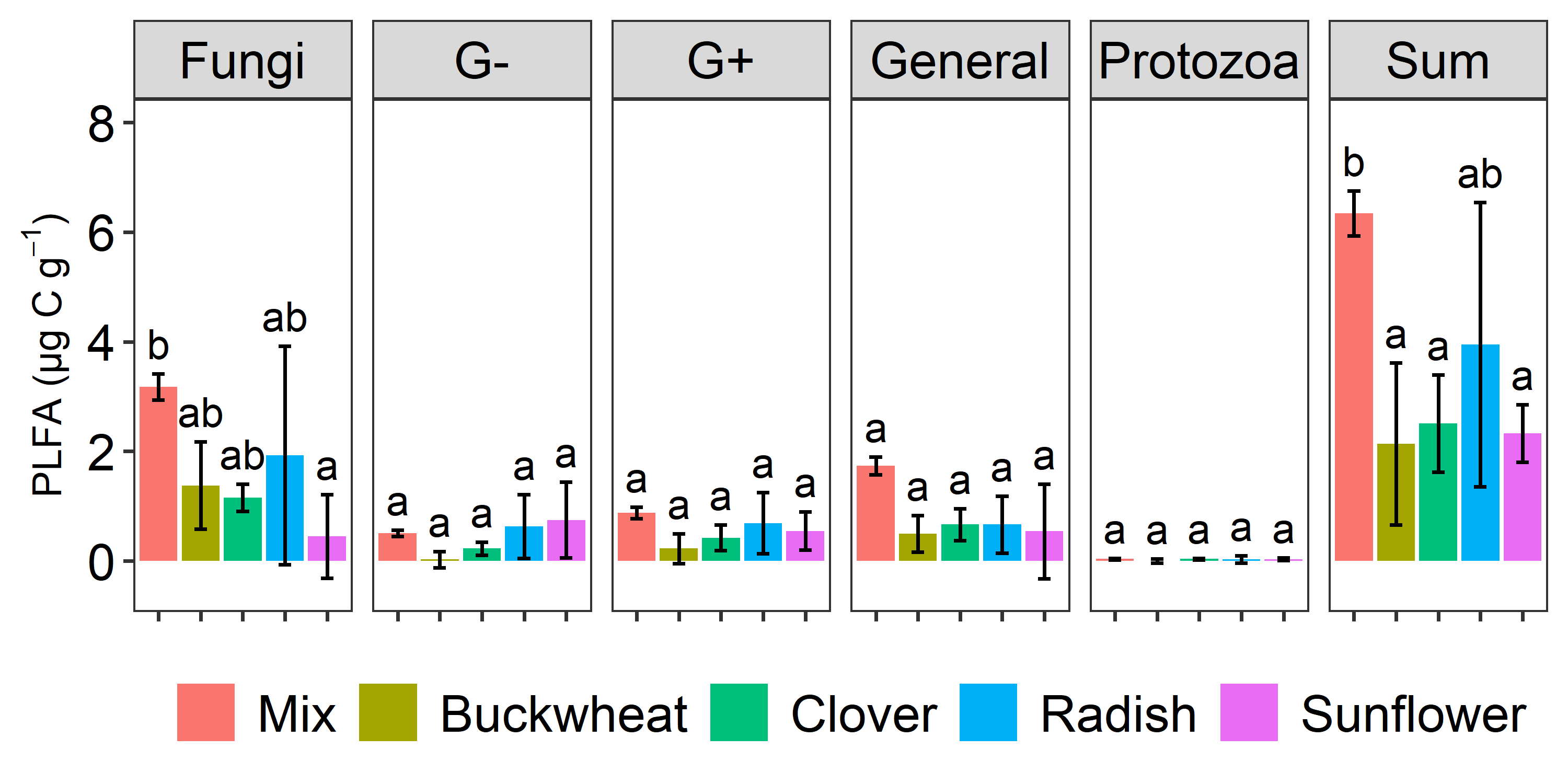


1. Crop residue- derived PLFA biomass in the mixture, and non-mixture (buckwheat, clover, radish, and sunflower) treatments. Sum is the sum of microbial biomass including all the microbial groups. G+, G-, and General represent Gram-positive, Gram-negative, and general bacteria. Error bars are standard deviations (n = 4). Different letters above bars indicate significant differences in PLFA biomass between treatments for that microbial group at *P* < 0.05.


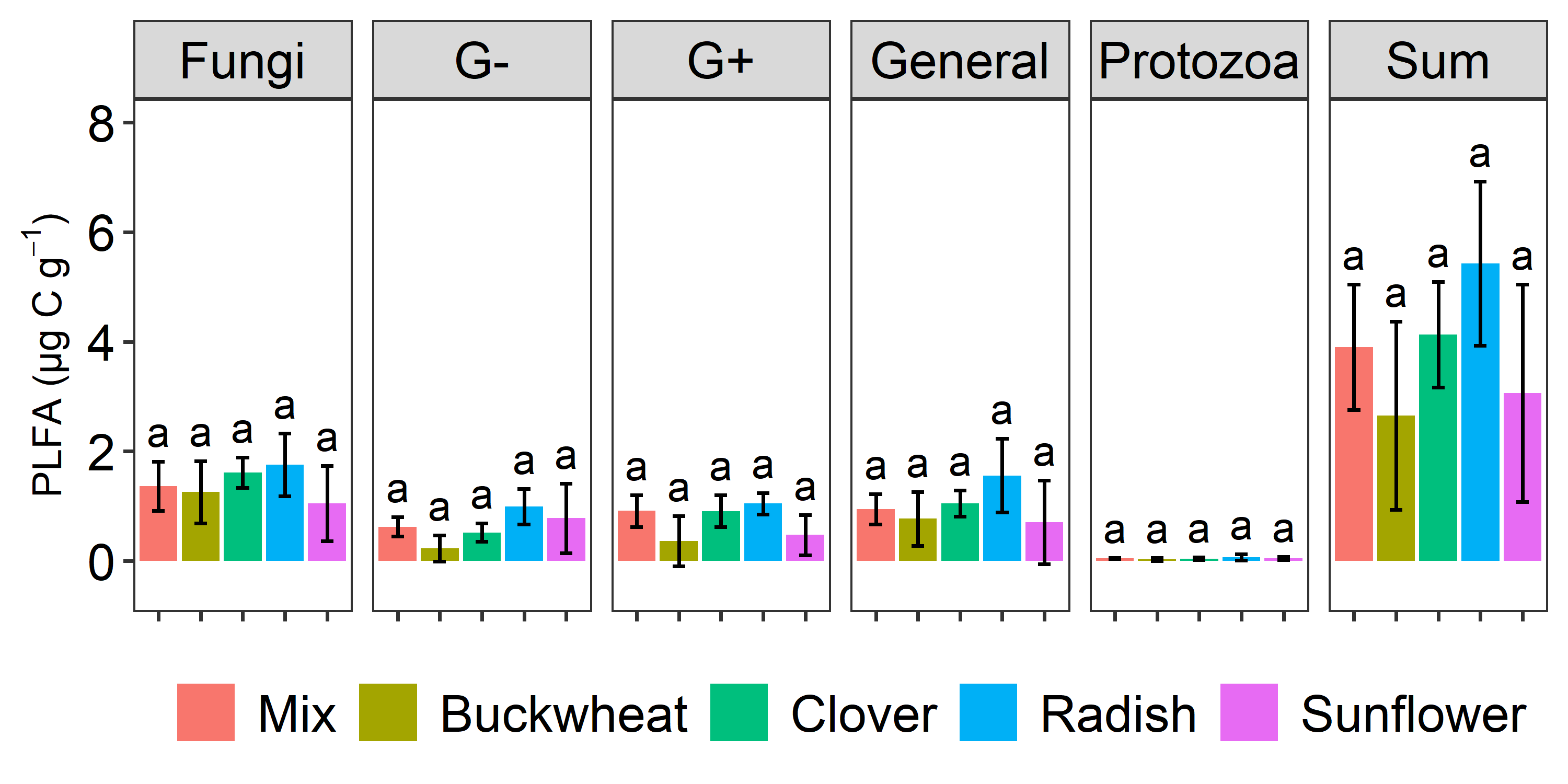


1. SOM (soil organic matter) derived PLFA biomass in the mixture, and non-mixture (buckwheat, clover, radish, and sunflower) treatments. Sum is the sum of microbial biomass including all the microbial groups. G+, G-, and General represent Gram-positive, Gram-negative, and general bacteria. Error bars are standard deviations (n = 4). Different letters above bars indicate significant differences in PLFA biomass between treatments for that microbial group at *P* < 0.05.

References

Buyer, J.S. & Sasser, M. 2012. High throughput phospholipid fatty acid analysis of soils. Applied Soil Ecology, 61, 127–130.

Jin, V.L. & Evans, R.D. 2010. Microbial 13C utilization patterns via stable isotope probing of phospholipid biomarkers in Mojave Desert soils exposed to ambient and elevated atmospheric CO2. Global Change Biology, 16, 2334–2344.

Mbuthia, L.W., Acosta-Martínez, V., DeBryun, J., Schaeffer, S., Tyler, D., Odoi, E., Mpheshea, M., Walker, F. & Eash, N. 2015. Long term tillage, cover crop, and fertilization effects on microbial community structure, activity: Implications for soil quality. *Soil Biology and Biochemistry*, **89**, 24–34.

Peacock, A.D., Macnaughton, S.J., Cantu, J.M., Dale, V.H. & White, D.C. 2001. Soil microbial biomass and community composition along an anthropogenic disturbance gradient within a long-leaf pine habitat. Ecological Indicators, 1, 113–121.

Wilkinson, S.C., Anderson, J.M., Scardelis, S.P., Tisiafouli, M., Taylor, A. & Wolters, V. 2002. PLFA profiles of microbial communities in decomposing conifer litters subject to moisture stress. *Soil Biology and Biochemistry*, **34**, 189–200.

Zelles, L. 1999. Fatty acid patterns of phospholipids and lipopolysaccharides in the characterisation of microbial communities in soil: A review. *Biology and Fertility of Soils*, **29**, 111–129.

Zhang, X., Xin, X., Yang, W., Zhu, A. & Ding, S. 2019. Short-term decomposition, turnover and retention of residue-derived carbon are influenced by the fertility level in a sandy loam soil. *Geoderma*, **349**, 68–78.

Zheng, Y., Wang, S., Bonkowski, M., Chen, X., Griffiths, B., Hu, F. & Liu, M. 2018. Litter chemistry influences earthworm effects on soil carbon loss and microbial carbon acquisition. *Soil Biology and Biochemistry*, **123**, 105–114.
